# Detection and Validation of Tow-Away Road Sign Licenses through Deep Learning Methods

**DOI:** 10.3390/s18124147

**Published:** 2018-11-26

**Authors:** Fabrizio Balducci, Donato Impedovo, Giuseppe Pirlo

**Affiliations:** Dipartimento di Informatica, Università degli studi di Bari Aldo Moro, 70125 Bari, Italy; fabrizio.balducci@uniba.it (F.B.); donato.impedovo@uniba.it (D.I.)

**Keywords:** tow-away sign, Italian road sign, pattern recognition, deep learning

## Abstract

This work presents the practical design of a system that faces the problem of identification and validation of private no-parking road signs. This issue is very important for the public city administrations since many people, after receiving a code that identifies the signal at the entrance of their private car garage as valid, forget to renew the code validity through the payment of a city tax, causing large money shortages to the public administration. The goal of the system is twice since, after recognition of the official road sign pattern, its validity must be controlled by extracting the code put in a specific sub-region inside it. Despite a lot of work on the road signs’ topic having been carried out, a complete benchmark dataset also considering the particular setting of the Italian law is today not available for comparison, thus the second goal of this work is to provide experimental results that exploit machine learning and deep learning techniques that can be satisfactorily used in industrial applications.

## 1. Introduction

The global economic crisis resulted in different consequences in some countries, leading to a rationalization of public spending and more careful resource management to face the lack of economic incomes for city communities, and often forcing public administrators to cut services or raise taxes. In this context, a tow-away (or driveway) sign is the set of objects for delimiting a private area (such as a car garage) that overlooks an open-to-public one suitable for stationing or moving vehicles; its goal is to prevent anyone (including the owner) from parking a vehicle in the demarcated area (set by law), allowing continuous moving into the private property.

**Figure 1:**
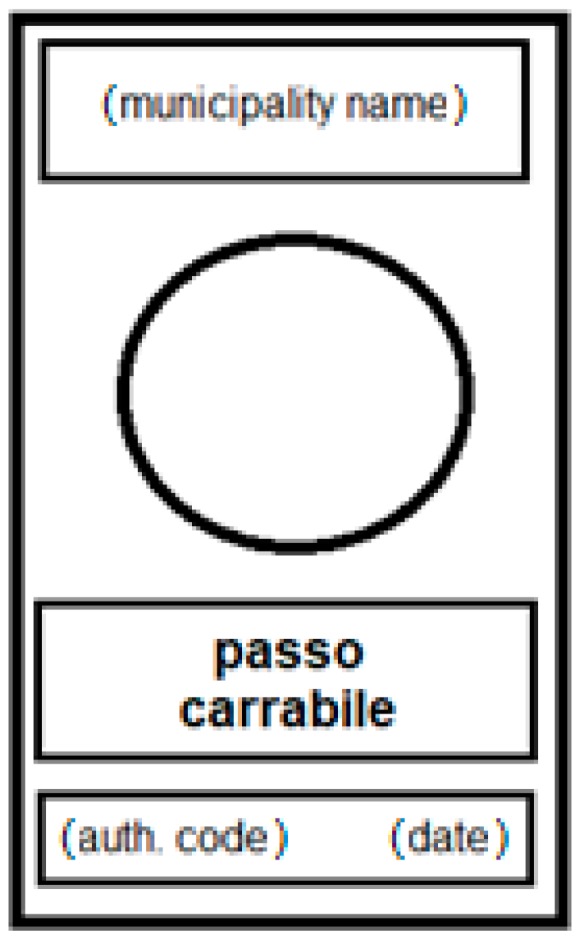
The pattern scheme of an Italian tow-away road sign.

Generally, and more specifically in Italy, a tow-away area is identified by a special sign that is put on the border between the private property and the public land; it has dimensions of 45 × 25 cm (or, when increased, of 60 × 40 cm) and it is composed in the upper band by the owner of the road (the municipality name), in the middle by the words, ‘passo carrabile’, together with a red prohibition symbol, while in the lower area, there is the authorization code and the issue date (a scheme is in Figure 1).

In countries where the transition from the manual management of the public utilities to a computerized one has not been completed and centralized, checking the validity of a license in good time becomes a problem that leads to delays and misunderstanding; also, when the paper records are not updated or there have been worker rotations. Another serious problem is the fraudulent and abusive use of such signs, with non-regulatory dimensions or with absent and counterfeited codes. Due to these reasons, the need of automatic systems that recognizes private tow-away road signs emerges, also to prevent citizens from having rights that are not due at the expense of others. It must also be considered that once a license has expired, until the new payment is made, the owner must remove this signal, and being able to contest the improper use of the signal in the missed period can be advantageous for the government.

The problem of recognizing figures and patterns has been dealt for a long time in the field of pattern recognition and artificial vision, with methodologies starting from the development of visual descriptors up to the classification using machine learning and deep learning techniques. In this work, a pre-trained model of a convolutional neural network will be exploited that automates the coding of visual features: In this way, our contribution can be identified primarily in the creation of the first manually annotated dataset built from real tow-away sign images. Secondly, it demonstrates that a dataset with such characteristics, after the fine-tuning of the state-of-art deep learning model, is sufficient to obtain interesting recognition performances.

The work is organized as follows: In Section 2, the related works are presented, Section 3 presents the system architecture and introduces the image dataset while in Section 4 the technologies and the employed algorithms are described. The experimental setup with results and comments is in Section 5 while, finally, in Section 6, there are conclusions and future works.

## 2. Related Works

The problem that this work faces is typical of pattern recognition and, more specifically, refers to object detection and classification where input data are images with a lot of variables (hardware and camera distortions, light changes, occlusions, distance, and so on) and, moreover, there is the license information consisting in printed strings made by literal and numerical patterns. In the literature, there are three main different approaches when approaching the object detection/classification related to the evolution of technologies and, moreover, belonging to hardware costs decreasing and to computational power increasing.

The first approach is related to hardware solutions and to the IoT (Internet of Things) paradigm, where small and cheap sensors and network systems are exploited. Berder, Quemerais et al. [1] propose a system for cooperative communication between vehicles and “intelligent” road signs exploiting autonomous radio communications while Katajasalo and Ikonen [2] have the same approach of a wireless identification, but using simple mobile devices, like smartphones and dedicated WLAN (wireless local area network). In Nejati [3] and in Guo et al. [4], systems designed via RFID (radio-frequency IDentification) and M-RFID (mobile RFID) are exploited to avoid traffic violations leading to car crashes, as done also by Jagriti et al. [5] while testing RFID readers installed on the underside of vehicle models, and by Paul et al. [6] when testing the distances between RFID sensors. With the goal of providing alternative paths to specific road signs, Quiao et al. [7] and Li et al. [8] focus on the ‘Stop’ road sign to reduce vehicle emissions by avoiding queues and unnecessary crossroad waiting.

The second approach refers to the exploit of classic pattern recognition and computer vision visual descriptors that during the time have been included in various software libraries. Lauziere et al. [9] propose a responsible system for identifying regions of interest (ROI’s) on color space labeling and connectivity. In Gil-Jimenez et al. [10], a classification based on comparison between the FFT (Fast Fourier transform) of the signature of a blob and the FFT of the reference shapes used in traffic signs emerges while a focus on the ‘Stop’ road sign is in Reference [11]. Carrasco et al. [12] execute a comparison between two methods used in the past for detection and recognition of road signs: Template matching and feed-forward neural networks while neural networks are also exploited by Miyata [13] for speed limit numbers recognition using an eigen space method and color features. Bose et al. [14] focus on enhanced dual-band spectral analysis in the hue-saturation-intensity (HSI) and RGB (Red Green Blue) domains while Marmo et al. [15], in 2006, enhanced identification of rectangular signs through the optical flow and Hough transform; Nguwi and Kouzani [16] present classification methods applied to road sign recognition divided into color-based, shape-based, and others, while Bui-Minh, Ghita et al. [17,18] face video detection and object occlusions.

In Krishnan et al. [19], a Bundle Adjustment improves the estimates obtained using triangulation by adjusting the camera’s pose estimate, and Belaroussi et al. [20] propose a case of study comparing results obtained by three algorithms, i.e., single pixel voting (SPV), contour fitting (CF), and pair-wise pixels voting (PWPV). Considering References [21,22], the detection, classification, and positioning with SIFT (Scale-Invariant Feature Transform) and bag of words (BoW) descriptors emerges while the purpose of Perry and Yitzhaky [23] is the understanding of road signs in vehicular active night vision through segmentation and illumination correction. Regarding the specific Italian context in which this work is concerned, there are noticeable results by Lombardi et al. [24]. A study comparing various visual descriptors is present in Russell and Fischaber [25] while Ding et al. [26] develop a system for detection and identification using the SURF (speeded-up robust features) [27] algorithm and GPGPU (general-purpose gpu) programming model. In Lin et al. [28], there is a hybrid approach based on adaptive image pre-processing models and two fuzzy inference schemes checking light illumination and the red color amount of a frame image.

Afridi et al. [29] use seven evolutionary models for the problem of road detection and identify vision features that enable a single classifier to successfully classify regions of various roads as opposed to training a new classifier for each road type; Bousnguar et al. [30] present a detection and recognition system, which first segments the image using a combination of RGB and HSV (hue saturation value) colors spaces and then searches for relevant shapes (circles, triangles, squares) using the Hough transform and corner detection with a support vector machine (SVM) while also Athrey et al. [31] implement an algorithm for traffic sign detection based on thresholding, blob detection, and template matching.

The machine learning approach, of which the deep learning paradigm results are more innovative and performing on videos and images, is the one exploited in this work and represents the evolution of the previous approaches abstracting the coding of visual features while demanding their detection to a series of convolutional filters (layers). One of the first approaches using convolutional neural networks (CNN) for road sign shape searching is in Adorni et al. [32] and in the work of Li and Yang [33], where deep Boltzmann machines are exploited for the road sign detection. Yang and Wang [34] propose an identification based on a learning wavelet for a directional multi-resolution ridgelet network (DMRRN) while Kouzani [35] exploits the ensemble learning that combines decisions of multiple classifiers.

Moreover, Hoferlin and Zimmermann [36] introduce local SIFT features for content-based traffic sign detection along with a technique called contracting curve density (CCO) to refine the localization of the traffic sign candidates. Islam et al. [37] also faced the recognition of Malaysian road and traffic signs from real-time videos coming from a camera on a moving vehicle by exploiting hybrid color segmentation and a multilayer artificial neural network. Overviews about road sign recognition and machine/deep learning are in Schmidhuber [38] and Stallkamp et al. [39], while in Huang et al. [40], an extreme learning machine (ELM) whose infrastructure is a single hidden-layer feed-forward network with histogram of gradient descriptors as features is employed. Filatov et al. [41] introduce the detection and recognition of traffic signs in real time a system based on a Raspberry Pi 2 and a webcam using color filters and morphological identification with a perceptron neural network. Vokhidov et al. [42] use CNN to recognize damaged arrow-road markings as input for an advanced driver assistant system (ADAS).

In Fulco et al. [43], CNN are used to classify specific German road signs, a task that resembles what was done in this work with the Italian ones; Hemadri and Kulkarni [44] develop a detector based on SVM [45] with the creation of an Indian benchmark signs dataset. Zhang et al. [46] use a probabilistic neural network to enhance visual features classification, and Borowsky et al. [47] and Cornia et al. [48] analyze eye movements to localize road signs and human behaviors in real-time driving while, finally, Abdi and Meddeb introduce in-vehicle augmented reality (AR) [49].

## 3. System Design and Image Dataset

To accomplish the specific tasks required and designed for a practical use, there are some constraints that are very important to be fulfilled by the Integrated System:

1. It must to be cheap and suitable for portable devices;
2. It must already be working and applicable on an industrial level without (a lot) of optimization;
3. It must to be easily used by non-IT skilled people (policemen and public officials) and mounted on official law enforcement vehicles in a transparent way;
4. It must provide the validation while interacting with public offices’ databases (also in real-time) as well as saving the image together with other meta-data (acquisition date, place, author).

The system will be calibrated and tested for the Italian context and, more specifically, in the typical urban environment formed by small or medium streets and large, but circumscribed, plazas. Assuming to mount the acquisition device on a police patrol car, the speed with which the acquisition hardware moves through the streets and its distance from the objectives are considered as variables that do not impact strongly on the results, as the police cars move in an orderly manner and with a speed harmonized to standard city traffic. Photos taken at a set time of only those with a pattern classified according to an acceptance threshold as a tow-away signal will be stored in the training dataset, also proceeding to identify its license validity.

**Figure 2:**
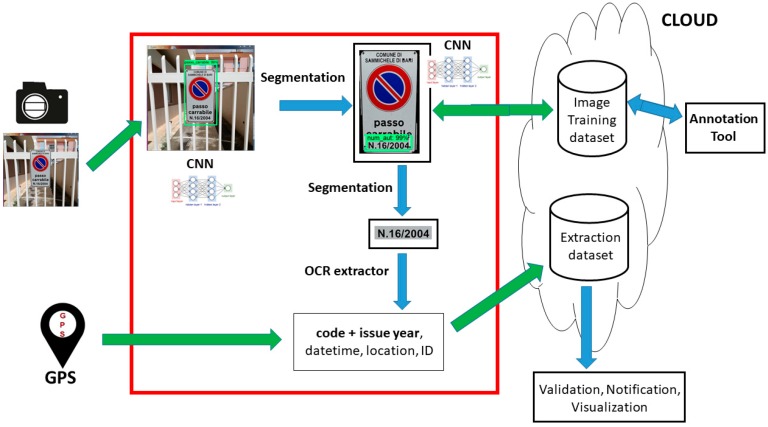
The proposed architecture for an integrated system. CNN = convolutional neural networks; OCR = optical character recognition; GPS = Global Positioning System.

The architecture in Figure 2 is implicitly divided into three sub-systems:

- Acquisition system: The hardware devices used for the images input, consisting of a portable photo/video recorder and a GPS (Global Positioning System) locator for the geographical metadata;
- Core system: Manages the raw input and exploits different technologies such as CNN, image segmentation, and optical character recognition (OCR) into a pipeline that accomplishes three tasks (object detection, pattern extraction, text extraction);
- Archive system: Constituted by an informative system (logically) dedicated to store the image dataset and the extracted metadata (sign code and year, date, time, location); moreover, there are two external modules dedicated respectively to the extracted data management (code validation, info visualization, alerts notification) and to the new images annotation through a visual tool.

This work is focused on the core system (components in the red square of Figure 1) and for this reason, the implementation aspects of the other two parts will be only mentioned.

### The Tow-Away Road Sign Image Dataset

**Figure 3:**
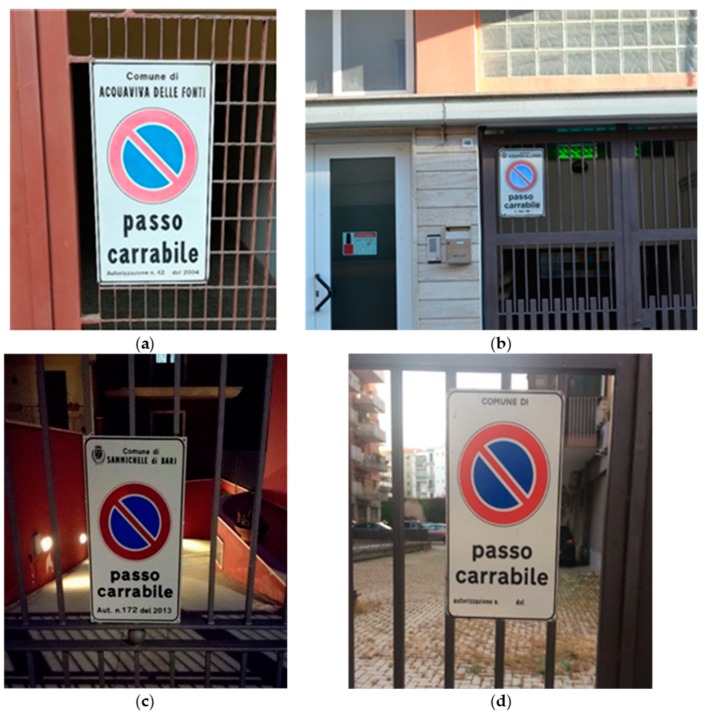
Examples from the classes of the tow-away road sign image dataset: (a) C1: Close distance and simple scenario; (b) far distance, complex scenario; (c) poor lighting; (d) false road sign or license code featuring missing/wrong information.

The acquisition and management of real images related to the tow-away road signs has the goal of building a dataset with which the machine learning component based on the convolutional model is trained and tested. In this way, they acquired and managed a total of 800 photos, divided into five different classes (Figure 3):

1. C1: 160 tow-away road sign closely photographed in a simple scenario;
2. C2: 160 tow-away road sign photographed from a distance, in a complex scenario with other elements (plants, machines, light poles, etc.);
3. C3: 160 tow-away road sign photographed in low light (photos taken in late afternoon/ evening);
4. C4: 160 tow-away road sign without authorization number and/or date and city name (a false license);
5. C5: 160 photos featuring a scenario without the tow-away road sign.

The image dataset is made up by five different image categories (also manually labeled as them) with the aim to train the convolutional model with the largest possible variety of the desired pattern so the final trained model will be able to recognize all the possible visual instances of a tow-away road sign pattern (also unseen before). Since the tow-away road sign pattern must be, by law, the same in the whole country, we can argue that the system will perform the same in each Italian city. What can affect the performances are the different environmental configurations (mountain, plain, hill, etc.) or dynamics (weather, speed, and acquisition inclinations, etc.), and for this reason, in the diagram of Figure 2, it is depicted a two-way arrow between the CNN model and the ‘image training dataset’, meaning that a continuous updating of the dataset triggers the periodic re-training of the model.

For each class, different hardware devices have been exploited to obtain three-pixel resolutions: Smartphone p8 Lite 2017 for the 2976 × 3968 resolution and the p9 Lite for the 3120 × 4160 resolution. A further device (reflex Nikon D3100) has been specifically used for the photos at the 4608 × 3072 resolution concerning the C2 class with the aim to simulate distant objects, but with a good level of detail.

In this way, the following sub-dataset(s) results:

1. D1: 2976 × 3968 photos:
  - 48 photos belonging to the C1 class;
  - 15 photos belonging to the C3 class;
  - 86 photos belonging to the C4 class;
  - 79 photos belonging to the C5 class.
2. D2: 3120 × 4160 photos:
  - 112 photos belonging to the C1 class;
  - 106 photos belonging to the C2 class;
  - 145 photos belonging to the C3 class;
  - 74 photos belonging to the C4 class;
  - 81 photos belonging to the C5 class.
3. D3: 4608 × 3072 photos:
  - 54 photos belonging to the C2 class.

## 4. System Development: Technologies and Algorithms

In this section the software, the models, and the algorithms used and customized to build a first prototype of the integrated system designed in Figure 2 are described, also explaining the reasons, merits, and defects of their use.

### 4.1. Tensorflow and Region-based Convolutional Neural Networks Model

A CNN is a model of a neural network architecture made by a succession of three-dimensional layers that represent filters on portions of an image, able to automatically recognize and extract heterogeneous visual features (angles, colors, gradients, shapes, etc.). The Region-based Convolutional Neural Network (R-CNN), not to be confused with a Recurren*t* Neural Network model (RNN), consists of three steps. In the first one, an image region is proposed, then its features are extracted, and, finally, all regions are classified according to their common features. Basically, it turns the object detection task into a problem of image classification. Since R-CNN models are very slow, their immediate descendant model (Fast-R-CNN) resembles the original in many ways but improves its detection speed by extracting features before proposing regions, so that performing only one CNN on the entire image instead of *n* CNN on over *n* overlapping regions.

TensorFlow is a machine learning library that supports a variety of applications and tasks, with a focus on deep learning and convolutional neural networks. It is a second-generation application programming interface (API) currently used by Google in both research and business products, such as speech recognition, Gmail, Google Photos, and Search. Tensorflow [50] has been released under the open source Apache 2.0 license on November 9th, 2015 and uses the data flow graph to represent all the computation and status in an automatic learning algorithm, including individual mathematical operations, parameters with their update rules, and input pre-processing. The data flow graph expresses the communication between the sub-computations explicitly simplifying the execution of parallel calculations: Each vertex represents a local computational unit and each edge represents the output from, or input to, a vertex while all data are modeled as tensors (*n*-dimensional matrices) with elements that have a small number of primitive types, such as int32, float32, or string; tensors naturally represent the inputs and results of common mathematical functions in many machine learning algorithms.

One of the utilities that TensorFlow offers for object recognition tasks is a pre-trained R-CNN model [51] that is responsible for defining the new state-of-art for classification and detection in large-scale visual recognition challenge: Its main hallmark is the improved use of computational resources that, thanks to a careful design, allow an increase of the depth and width of the graph network while keeping the computational budget constant. Over the years, different versions of this model have evolved, with the aim of reducing the error rate in the test phases.

The R-CNN model used the COCO (common objects in context) set for its pre-training. It is a large image dataset designed for object detection, segmentation, detection of key points, and generation of captions by exploiting annotations. Image objects as patterns are labeled using the segmentation to facilitate the precise pixel location: The dataset contains photos of 91 classes that would be recognizable and, with a total of 2.5 million instances labeled in 328,000 images, the COCO projects permits pre-training of a CNN model that can be customized and specialized by further training on new classes and object patterns.

### 4.2. Image Segmentation

This algorithm must extract all the areas of interest identified in the input image from the two detectors via the R-CNN model. Our system will be used the first time to extract the entire tow-away road sign pattern and the second one to extract the sub-area containing the printed text of the license information (code and date).

The method extracts foreground objects from an image requiring little iteration since the only input required is to draw a bounding box around the target object that is the R-CNN input since the training images are labeled and the output on the test ones is obtained in the form of box coordinates.

The steps are:

1. Take as input the foreground, the background, and the unknown part of the image that may be in the foreground or in the background. This is normally done by selecting a rectangle around the object of interest and marking the region within this rectangle as unknown. The pixels outside this rectangle are marked as a ‘known background’;
2. Create an initial segmentation of the image where unknown pixels are placed in the foreground class and all known background pixels are classified as backgrounds;
3. The foreground and the background are modeled using the Gaussian Mixture Models (GMMs) in Equation (1);
4. Each foreground pixel is assigned to the most probable Gaussian component in the GMM in the foreground and the same process is done with the pixels in the background, but with GMM components in the background;
5. The new GMMs are learned from the pixel sets that were created in the previous steps;
6. A graph chart is created and a graph cut is used to find a new classification of pixels both in the foreground and in the background;
7. Steps 4–6 are repeated until the classification converges.

(1) Fα,µ,σ2(X) = ∑j=1mαj12πσje−(x−µj)22σj2.

### 4.3. Optical Character Recognition Extractor

In this work, the OCR module is used in the final part of the whole system to simulate the license validation task by extracting from the tow-away road sign the printed area with the code that has to be compared with the official one registered in an external database.

Most of the techniques on which optical character recognition systems are based are borrowed from pattern recognition and image processing focusing on specific object classes, such as letters, digits, and special symbols, for punctuation and formatting.

A typical OCR system consists of several components, starting from the source (digitizing the analog document) and from the localization of regions, where each symbol is extracted through the segmentation process; they must be pre-processed, cleaned by background noises through filters, and normalized by size, inclination, and rotation. The identity of each symbol is found by comparing the features extracted with descriptors of symbol classes, obtained through previous learning, template matching, statistical techniques, or dictionaries. Finally, the information is used to reconstruct words and numbers of the original text by grouping them and correcting errors.

The pipeline to extract information from an image is as follows:

1. Adaptive thresholding that converts the image into a binary version;
2. Analysis of the page layout to extract the blocks of the document;
3. Detection of the baselines of each line and division of the text into pieces using spaces;
4. Characters are extracted from the words and the text recognition is performed in two steps. In the first, using the static classifier each word found is passed to an adaptive classifier as training data; later, the second pass is performed on the whole page using the newly learned adaptive classifier, where words that have not been recognized well enough are recognized again.

## 5. Metrics, Experiments, and Results

The acquisition of the tow-away road signs dataset is a necessary, but not sufficient, condition to execute the training of the R-CNN model of the system: Since it is exploited, a supervised machine learning technique, they require labeled examples (objects into the images) to train a model that must learn to discriminate or generate new examples based on those previously seen.

### 5.1. Performance Evaluation Metrics

**Figure 4:**
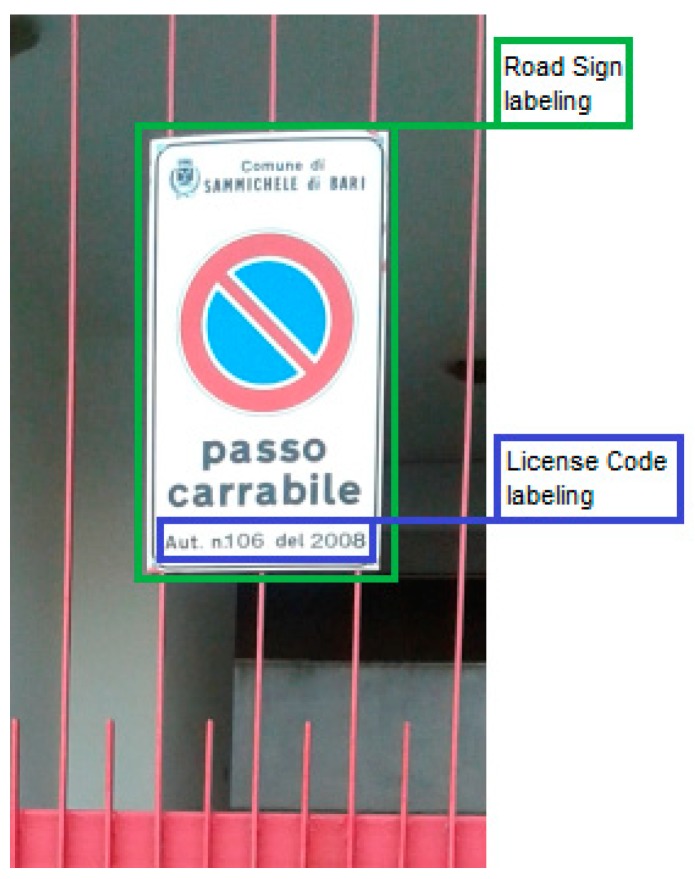
The bounding boxes drawn by a human annotator to build the ground truth image dataset.

Each image of the dataset has been annotated by humans (Figure 4) to create a ground truth dataset, where a green square identifies the road sign area and a blue square identifies the license code area: In this way, for each image, an XML file will be created containing the bounding boxes description from which the R-CNN model learns ´what to look’, i.e., the location where is placed the patterns of interest that must to be identified and extracted from other images.

With this image annotation, the Jaccard Index or Intersection over Union can be exploited, an evaluation metric calculated for a recognition task. It measures the road sign recognition rate by comparing the original ground truth bounding box area (named A) (obtained from the manual annotation with XY pixel coordinates) with a new one (named B) predicted by the model.

In Equations (2) and (3), the numerator represents the overlapping area while the denominator is the joining one, i.e., the total area made by both the boxes. Once this value has been calculated, it can be considered a value greater than a threshold, *t*, as an index of a good forecasting performance.

(2) J(A,B)= |A∩​B||A∪​B|.

Considering A and B as two-pixel areas linearized as vectors, **A** = {$x_{1}$,$x_{2}$,…,$x_{n}$} and **B** = {$y_{1}$,$y_{2}$,…,$y_{n}$}, then:

(3) J(A,B)=∑i=1nmin(xi,yi)∑i=1nmax(xi,yi).

Other metrics exploited on the image pixels that, in this work, will be used to measure the system global detection performance on the test set after the training will be:

Accuracy = (TP + TN)/(TP + TN + FP + FN) (4)

Precision = TP/(TP + FP) (5)

Recall = TP/(TP + FN) (6)

f1−score = 2 × (Precision × Recall)/(Precision + Recall) (7)

where each variable is a counter and:

- TP are true positive cases, road sign present in the image and detected by the system;
- TN are true negative cases, road sign not in the image and not detected by the system;
- FP are false positive cases, road sign not in the image, but detected by the system;
- FN are false negative cases, road sign present in the image, but not detected by the system.

If the object recognition output (R-CNN accuracy) overcomes a threshold, t∈I, *0 ≤ I ≤* 1 (*t* = 0.9 for example), then the proper case counter is increased; this metric will be used for the evaluation of both the tow-away road sign and the license information (code number and date) detection.

The previous metrics give a global and absolute evaluation of the system for the object recognition and classification tasks (measuring ‘if’ the object is present), but it is also useful to evaluate the Global Probabilistic Score that tells ’how much‘ the object has been recognized (i.e., no longer considering the threshold value).

In this way, for each class *C* of the dataset, from (8), the value $P_{s}$ as the mean for the accuracy score $p_{i}$ calculated for the i-th input image will be calculated:

(8) $$P_{s}\left(C\right)=\frac{\sum_{i=1}^{n}p_{i}}{n}$$

### 5.2. Experimental Design

To validate the system measuring its performances, it is useful to consider it as composed by two separated detectors (road sign pattern and license information) deploying a K-Fold Cross Validation design for their training (and testing), with K = 4, where the dataset is split into K equally numerous folders.

In four iterations, three folders are used in turn for training while the left one is for the testing, with the aim to optimize the model internal parameters and weights. In each of the four iterations, the R-CNN model will cycle on the training dataset for *m* times since it does not process its input one-at-a-time, but to increase the throughput, arranges data in blocks of a certain size (batch size value).

**Figure 5:**
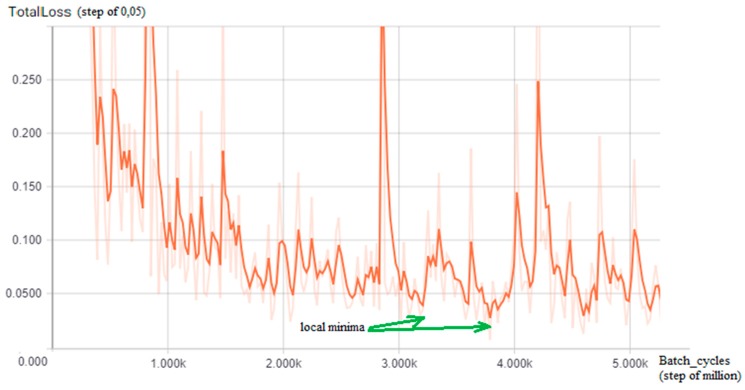
The TensorBoard graph of the loss function (y-axis, step of 0,05) on a subset of the training dataset for the road sign detector: Interesting local minima for the number of training batch cycles results in approximately 3,200,000 and 3,800,000 (green arrows, x-axis = batch size cycle amount, step of million).

**Figure 6:**
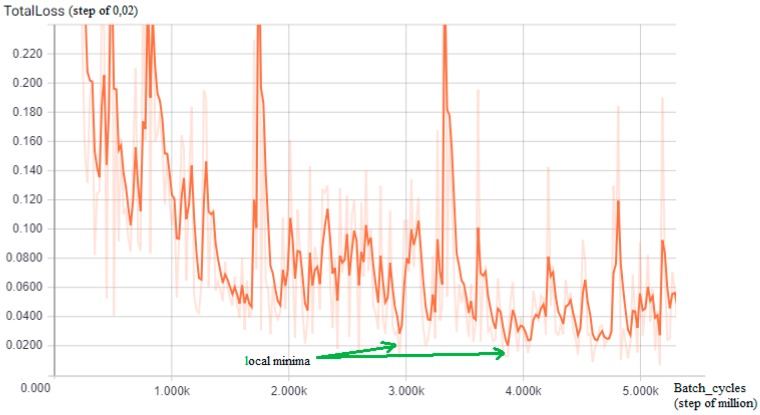
The TensorBoard graph of the loss function (y-axis, step of 0,02) on a subset of the training dataset for the license code area detector: Interesting local minima for the number of training batch cycles results in approximately 2,950,000 and 3,900,000 (green arrows, x-axis = batch size cycle amount, step of million).

The training of a CNN is a very time-consuming activity even when using a pre-trained model, which retains its original weights about the classes, and updates them with new images in the training set; since, in this work, a Tensorflow R-CNN is adapted to guess how many batch cycles are required to reach an acceptable loss value, the TensorBoard tool has been used: By performing a preliminary training phase on a subset of the entire dataset (35%), a graph of the loss function identifying oscillating trends has been visualized, but with local and global minima (green arrows) for each of the two detectors (Figure 5 and Figure 6) from which comes that in the case of the first detector (tow-away road signs), the value to choose is 3,200,000, reaching a minimum loss of 0 048 while for the second detector (license code information), the batch value chosen is 2,950,000, reaching the minimum loss of 0.025.

Finally, a new class, ‘Italian tow-away’, has been added to the convolutional model so that, in its last output layer, it will be considered a new output class related to the pattern on which it was trained and specialized with the ground truth dataset built in this work.

### 5.3. Experiment Execution and Discussion

To perform the experimental phase, the three functional modules (Road Sign detector, License Info detector, OCR string extractor) have been managed separately since the amount of training images change between them and, above all, we were interested in highlighting each other’s strengths and weaknesses; moreover, s0 represents the first detector (R-CNN) at its initial step, i.e., without the specific training for the tow-away road sign using the new images of our specific ground truth dataset.

For the Road Sign detector training, each of the K = 4 fold was made up of 160 images, divided proportionally to the five categories mentioned above, for a total of 640 images, since images that did not contain tow-away road signs (without any annotation) were left out. For the License info detector training, each fold was made up of 120 images, divided proportionally to the categories mentioned above, for a total of 480 images, leaving out images that not only did not contain tow-away road signs, but also lack the printed string area annotation. Results for the global performance of the two detectors, employing all the previous metrics, are in Table 1 (threshold values for the detectors are, respectively, 0.90 and 0.95).

The detection results are all over 96%, with a weak supremacy of the License detector over the Sign one (only the Recall 98.4% is in favor of the first one); the two f1-scores are very near (98% and 98.4%) while the License precision reaches over 99%.

For the OCR extractor global results, considering that all the false road signs have been removed from the training dataset (and not being interested to annotate invalid string areas), the results are expressed in the form of ‘recognized’ (425) and ‘not recognized’ (55), simulating a matching between the extracted strings and code with the real one contained in a database. The final result for the simulation of the Validation task is (425/480) × 100 = 88.5%.

#### Probabilistic Score

Results for the Probabilistic score with cross validation mode are in Table 2 from which it comes that, for the two detectors, results are always over 94.8% for each test folder of the K = 4 training cycles, and over 86.5% for the OCR extractor. Sign reaches a mean of 97.4% while License (that in the working pipeline comes after the first detector) has a mean of 96.6%. The third stage of the system results are a little weaker since the string translation from the OCR extractor reaches a global score of 88.5%, deviating by about 8% from the other two.

Considering the different sub-datasets about image pixel resolution, results are in Table 3, Table 4 and Table 5. 

Taking the three system modules (plus the not-trained detector), it is possible to see that *s0* is totally unable to recognize the pattern (new class) of the Italian tow-away road sign, always below 10%, above all on long distance images (0.13%); globally, considering the whole dataset, the system results are more confident in recognizing images from C1 with a mean of 73.5% followed by C3 (72.8%) and with equal scores between C2 and C4 (69.7%).

After the new training, Signal reaches over 97.5% on each pixel resolution, with excellent results and strong increase on images at a further distance and higher resolution (from 0.13 to 99%). For each resolution, License has a performance a little lower than Signal (between 0.05% and 1%), but it is OCR that determines the decrease in performance in the final step of the system (from −6.36% to −8.83%).

When tested on images where there is no pattern of a road sign, the system is never wrong (100%), but this result suggests an increase of the images of class C5 trying to ’deceive‘ the system with similar or false patterns; in the 2976 × 3968 sub-dataset, the best-recognized class is C3 (variable lightness, 73.69%) while in the 3120 × 4160 resolution, it is C1 (close distance and normal lighting, 73.99%), the most recognized one.

## 6. Conclusions and Future Work

This work presents the design and the development of an intelligent system aimed at solving a practical goal with social purposes of fairness and social equality, by exploiting innovative pattern recognition and machine learning techniques. It has been shown how the reuse of a powerful deep learning R-CNN model specialized in object recognition, together with a specific training image dataset constituted by instances of Italian tow-away road signs, performs noticeable experimental results, leading to an improvement of over 90% (from a model with generic training to a model after the specific training with our dataset). Moreover, the two detectors reach probabilistic scores with a mean over 98% while the OCR surpasses the 88% of textual well-recognized extraction.

Although very interesting results have been obtained during this first phase, the improving of the detectors is required, and, in particular, of the OCR one, to reduce the error rates in complex scenarios, also considering the real-time image acquisition (and/or classification) at different speeds while exploiting heterogeneous hardware devices or IoT platforms, like Arduino or an NVIDIA Jetson TX one as seen in [52]. In this way, the dataset expansion and refinement, together with a stage of data augmentation and with alternative acquisition modes (‘Google Street View’ or surveillance cameras with video analysis), could be very interesting ways to pursue, together with experimentation in Italian locations different from that in which the dataset has been developed, to verify its stability and generality.

**Figure 7:**
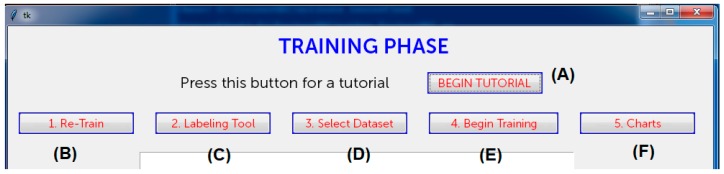
The prototype of an interactive user-interface for the propose system (functions described with characters A–F in brackets).

Referring to the architecture in Figure 2, there are other modules to develop, from the annotation/visualization tools to the information management and validation ones: Considering that this system will be used by no-IT people, specific user-interfaces and usability studies must be carried out (a piece of the prototype for an interactive user-interface to execute the tutorial (A), the training (B,D,E), the labeling (C), and the charts tool (F) is in Figure 7). Other specific modules deal with retraining strategies during the lifecycle of the application [53,54].

Finally, from the point of view of the services offered to citizens by a public administration, it would be very interesting for an on-line application that by sending a picture from a simple smartphone permits to pay directly the fee for the renewal of the license without any further annoyance and loss of time and also enhancing the image training dataset.

## Figures and Tables

**Table 1:** Metrics for the performance results of the three modules.

	TP	TN	FP	FN	Accuracy	Precision	Recall	f1-score
Road Sign det.	616	0	14	10	96.25	97.78	98.40	98.09
License Info det.	465	0	4	11	96.88	99.15	97.69	98.41

TP = true positive cases, TN = true negative cases; FP = false positive cases; FN = false negative cases.

**Table 2:** Results for the Probabilistic Score of the three modules.

	Road Sign Det.	License Info Det.	OCR Extr.
Fold 1	95.3	96.3	89.2
Fold 2	97.8	98.1	90.8
Fold 3	98.3	97.2	87.5
Fold 4	98.3	94.8	86.6

**Table 3:** Results for the Probabilistic Score on the 2976 × 3968 image pixel resolution.

	s0	Road Sign Det.	License Info Det.	OCR Extr.	Mean
C1	10.23	99.00	96.85	85.85	72.98
C2	--	--	--	--	--
C3	2.58	99.00	98.95	94.25	73.69
C4	13.48	96.73	99.28	--	100,00
C5	--	100,00	--	--	100,00
Mean	8.76	98.68	98.36	90.05	

**Table 4:** Results for the Probabilistic Score on the 3120 × 4160 image pixel resolution.

	s0	Road Sign Det.	License Info Det.	OCR Extr.	Mean
C1	7.95	97.25	98.05	92.73	73.99
C2	0.25	96.03	90.45	80.20	66.73
C3	1.40	96.90	98.95	90.40	71.91
C4	11.75	97.78	98.90	--	69.48
C5	--	100,00	--	--	100,00
Mean	5.34	97.59	96.59	87.78	

**Table 5:** Results for the Probabilistic Score on the 4608 × 3072 image pixel resolution.

	s0	Road Sign Det.	License Info Det.	OCR Extr.	Mean
C2	0.13	99.00	98.95	92.60	72.67
